# Predictive implications of albumin and C-reactive protein for progression to pneumonia and poor prognosis in *Stenotrophomonas maltophilia* bacteremia following allogeneic hematopoietic stem cell transplantation

**DOI:** 10.1186/s12879-017-2745-6

**Published:** 2017-09-22

**Authors:** Kaito Harada, Noritaka Sekiya, Tatsuya Konishi, Akihito Nagata, Yuta Yamada, Toshiaki Takezaki, Satoshi Kaito, Shuhei Kurosawa, Masahiro Sakaguchi, Shunichiro Yasuda, Shugo Sasaki, Kosuke Yoshioka, Kyoko Watakabe-Inamoto, Aiko Igarashi, Yuho Najima, Takeshi Hagino, Hideharu Muto, Takeshi Kobayashi, Noriko Doki, Kazuhiko Kakihana, Hisashi Sakamaki, Kazuteru Ohashi

**Affiliations:** 1 Division of Hematology, Tokyo Metropolitan Cancer and Infectious Diseases Center Komagome Hospital, Tokyo, Japan; 2 Department of Infection Prevention and Control, and Department of Clinical Laboratory, Tokyo Metropolitan Cancer and Infectious Diseases Center Komagome Hospital, 3-18-22 Honkomagome, Bunkyo-ku, Tokyo, 1138677 Japan

**Keywords:** Albumin, C-reactive protein, *Stenotrophomonas maltophilia*, Hematopoietic stem cell transplantation

## Abstract

**Background:**

*Stenotrophomonas maltophilia* (*S. maltophilia*) bacteremia causes significant morbidity and mortality in immunocompromised hosts. However, incidence and risk factors for mortality in *S. maltophilia* bacteremia following allogeneic hematopoietic stem cell transplantation (allo-HSCT) remain controversial. The primary aim of this study is to clarify factors associated with poor prognosis of allo-HSCT recipients with *S. maltophilia* bacteremia.

**Methods:**

From January 2005 to December 2014, patients with hematological diseases and *S. maltophilia* bacteremia at a single transplantation center in Japan were examined for incidence and 90-day mortality. Prognostic factors associated with 90-day mortality among allo-HSCT recipients were analyzed by log-rank test, and significant variables in the univariate analysis were included in the multivariate Cox proportional-hazards regression model.

**Results:**

A total of 65 patients, including 47 patients undergoing allo-HSCT, developed *S. maltophilia* bacteremia. The incidence of *S. maltophilia* bacteremia was significantly higher in allo-HSCT recipients compared to patients not receiving allo-HSCT (6.53 vs. 0.36 per 100 admissions, respectively; *p* < 0.01). The overall 90-day mortality in allo-HSCT recipients was 43%. Independent risk factors for 90-day mortality were low serum albumin (<3.0 g/dl) (HR = 10.86; 95% CI, 3.27–36.12) and high serum C-reactive protein (CRP) (≥10.0 mg/dl) (HR = 3.28; 95% CI, 1.00–10.72). Among 9 patients with both high CRP and low albumin, 5 had pneumonia at the onset of bacteremia and the remaining 4 patients developed pneumonia in a median of 3 days (range, 1 to 8 days) even under effective treatment. All 9 patients eventually died in a median of 2 days (range, 2 to 32 days). The probabilities of developing pneumonia in patients with or without high CRP and low albumin levels were 100% (9/9) and 10.5% (4/38), respectively (*p* < 0.01).

**Conclusions:**

Allo-HSCT recipients had higher rates of *S. maltophilia* bacteremia than did patients not receiving allo-HSCT. High serum CRP and low serum albumin at the onset of bacteremia are predictive of disease progression to pneumonia and poor prognosis.

**Electronic supplementary material:**

The online version of this article (10.1186/s12879-017-2745-6) contains supplementary material, which is available to authorized users.

## Background

*Stenotrophomonas maltophilia* (*S. maltophilia*) is a nosocomial, aerobic, glucose non-fermentative gram-negative bacillus that can be isolated from natural or hospital environments [1–3]. Although *S. maltophilia* possesses a limited pathogenicity, the organism can cause serious infections in immunocompromised hosts, especially in hematopoietic stem cell transplantation (HSCT) recipients [3, 4].

As allo-HSCT recipients are a severely immunocompromised population with complex comorbidities, these individuals are more likely to have multiple risk factors and to develop fatal infections such as hemorrhagic pneumonia than are patients receiving chemotherapy or autologous (auto)-HSCT [3, 5–8]. Several studies have reported predisposing risk factors for *S. maltophilia* infection among allo-HSCT recipients; these factors include prolonged neutropenia, existence of graft-vs.-host disease (GVHD), indwelling central venous catheters (CVC), urinary catheters, prior treatment with broad-spectrum antibiotics, prolonged hospitalization, diarrhea, and severe mucositis [3, 7–9]. Therefore, data for incidence, morbidity, and mortality in allo-HSCT recipients should be analyzed separately from those in patients not receiving allo-HSCT. However, few studies have examined the precise incidence rate of *S. maltophilia* bacteremia in allo-HSCT recipients, despite the increasing incidence of this infection in heterogeneous populations including allo-HSCT recipients and patients receiving chemotherapy or auto-HSCT [10–12]. Additionally, past studies have revealed that profound neutropenia, septic shock at the onset of bacteremia, pneumonia, inappropriate antimicrobial therapy, and presence of CVCs were risk factors for mortality of *S. maltophilia* bacteremia in heterogeneous groups [5, 6, 11–13]. Nevertheless, risk factors for mortality focusing on allo-HSCT recipients have not been investigated in depth.

The objective of this study is to investigate the incidence, clinical characteristics, treatment outcome, and risk factors for mortality of allo-HSCT recipients with *S. maltophilia* bacteremia in a single bone marrow transplantation center in Japan over a 10-year interval.

## Methods

### Patients

From January 2005 to December 2014, data on demographic and clinical characteristics, laboratory data, antimicrobial susceptibility, treatment, and outcome were collected for all *S. maltophilia* bacteremia cases observed at an 800-bed tertiary care hospital with a 32-bed transplantation ward. All cases identified in patients with hematological diseases were included. This study was approved by the Ethics Review Committee in our hospital.

### Definition and microbiology

*S. maltophilia* bacteremia was defined as at least one positive blood culture of *S. maltophilia* with clinical signs of infection. Initial source of infection consisted of central line-associated blood stream infection (CLABSI), pneumonia, cellulitis, and sources unknown. CLABSI was diagnosed when *S. maltophilia* was detected from the CVC tip with a positive blood culture, or according to the widely accepted definition of differential time to positivity [14]. Pneumonia and cellulitis were defined as active clinical signs and symptoms with newly detected imaging findings consistent with the site of infection, with or without isolation of the organism from concomitant specimens [11, 15]. The BacT/Alert 3D (bioMérieux, France) automated blood culture system was used. Identification and antimicrobial susceptibility testing of isolates also were conducted using the MicroScan Walkaway plus System (Beckman Coulter, Inc., USA) based on Manual M100-S23 of the Clinical and Laboratory Standards Institute. Polymicrobial, continuous, and breakthrough bacteremia were defined as follows: bacteremia due to multiple organisms including *S. maltophilia*; bacteremia due to *S. maltophilia* that was detected persistently in the same patient; bacteremia in patients receiving appropriate therapy for the microorganism that was grown from the blood [11, 16]. Effective antimicrobial therapy was defined as the use of one or more agent active against *S. maltophilia* in an adequate dose [17]. Severe sepsis and septic shock were defined by the SSCG2012 criteria [18]. *S. maltophilia*-related mortality was judged by a review of the medial chart. Patients who succumbed to *S. maltophilia* bacteremia or complications of the bacteremia, not primary hematological disease itself, were judged as *S. maltophilia*-related deaths. In the analysis of *S. maltophilia*-related mortality, patients who died, but were not judged as *S. maltophilia*-related deaths, were censored on the date of death. Neutropenia was defined as an absolute neutrophil count (ANC) less than 0.5 × 10^9^/L. Profound and prolonged neutropenia were defined as an ANC less than 0.1 × 10^9^/L and less than 0.5 × 10^9^/L for more than 14 days, respectively. Neutrophil engraftment was defined by an ANC of at least 0.5 × 10^9^/L for 3 consecutive days. Adverse events, such as diarrhea and mucositis, were graded according to the National Cancer Institute Common Terminology Criteria for Adverse Events, version 4.0. Conditioning intensity was classified as myeloablative or reduced-intensity conditioning according to Center and International Blood and Marrow Transplant Research classification [19]. Our policies of antimicrobial prophylaxis in allo-HSCT were as follows: levofloxacin for bacterial infections; fluconazole, itraconazole, or voriconazole for fungal infections; trimethoprim-sulfamethoxazole (TMP-SMX) or pentamidine inhalation for *Pneumocystis jirovecii* pneumonia; and acyclovir for viral infections. Cefepime, tazobactam-piperacillin, or meropenem was administered for empirical treatment of febrile neutropenia according to the IDSA guidelines [20].

### Statistical analysis

We compared allo-HSCT recipients with patients not receiving allo-HSCT to investigate the incidence, clinical characteristics, and mortality. Patients not receiving allo-HSCT were consisted of those with non-malignant hematological disorders, and those who received chemotherapies or auto-HSCT. Fisher’s exact test for categorical variables and the Mann-Whitney U-test for continuous variables were used. Prognostic factors associated with 90-day mortality among allo-HSCT recipients only were analyzed by log-rank test according to age, sex, C-reactive protein (CRP), albumin, creatinine, neutropenia, onset of bacteremia, existence of acute GVHD, primary disease risk, presence of polymicrobial bacteremia, severe sepsis or septic shock, initial source of infection, removal of CVC, source of transplantation, related or unrelated source, HLA mismatching, conditioning intensity, sex mismatching, antibody of cytomegalovirus in recipients, ABO mismatching, and study period. The serum values of CRP, albumin, and creatinine were assessed at the onset of bacteremia. The cut-off values of albumin and CRP were determined using Receiver Operating Characteristics curve analysis (Additional file 1: Figure S1). Significant variables in the univariate analysis were included in the multivariate Cox proportional-hazards regression model with stepwise method, and both hazard-ratios and 95% confidence intervals (95% CI) were calculated. Two-tailed *p*-values of less than 0.05 were considered significant. All statistical analyses were conducted using EZR software [21].

## Results

### Incidence of *S. maltophilia* bacteremia

**Fig. 1:**
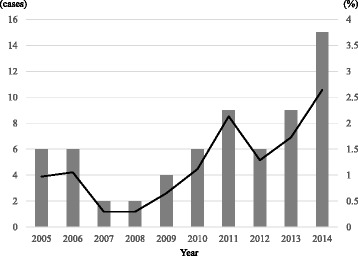
Cases and incidence of *S. maltophilia* bacteremia in Komagome Hospital patients with hematological diseases from 2005 to 2014

There were 5679 admissions and 720 allo-HSCTs with 191,090 patient-days during the study period. A total of 65 patients with *S. maltophilia* bacteremia were identified, with the incidence of 1.14 cases per 100 admissions and 3.40 cases per 10,000 patient-days. The incidence of *S. maltophilia* bacteremia was significantly higher in allo-HSCT recipients compared to patients not receiving allo-HSCT (6.53 vs. 0.36 per 100 admissions, respectively; *p* < 0.01, and 6.19 vs. 1.56 per 10,000 patient-days, respectively; *p* < 0.01). Notably, the overall incidence of *S. maltophilia* bacteremia increased during the latter 5-year period compared to the former 5-year period (1.79 vs. 0.63 per 100 admissions, respectively; *p* < 0.01; Fig. 1). Among the 720 allo-HSCT cases during the study period, there were no significant differences in the incidence of *S. maltophilia* bacteremia between each stem cell source, 7.3% (34/468) in bone marrow, 4.4% (7/158) in peripheral blood, and 6.3% (6/94) in cord blood (*p* = 0.42).

### Patient characteristics

**Fig. 2:**
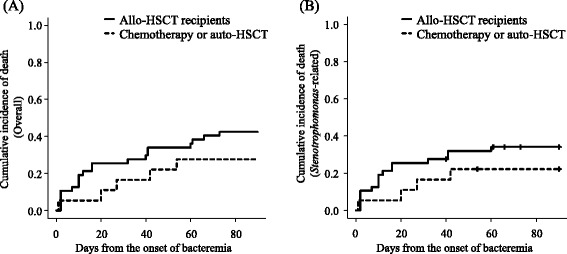
Overall (**a**) and *S. maltophilia*-related mortality (**b**) in allo-HSCT recipients or in patients not receiving allo-HSCT. A vertical line (|) indicates censoring from the analysis

Of all patients with *S. maltophilia* bacteremia, 47 (72.3%) had received allo-HSCT. Compared with patients not receiving allo-HSCT, allo-HSCT recipients were younger (median; 49 vs. 59 years old; *p* = 0.02), and more likely to have diarrhea (55.3% vs. 16.7%; *p* < 0.01), mucositis (42.6% vs. 5.6%; *p* < 0.01), CVC (95.7% vs. 61.1%; *p* < 0.01), total parenteral nutrition (44.7% vs. 5.6%; *p* < 0.01), and insulin therapy within 7 days (27.7% vs. 0%; *p* = 0.01) (Table 1). Although the overall 90-day mortality rate in allo-HSCT recipients was nominally higher (42.6% [20/47] in allo-HSCT vs. 27.8% [5/18] in non-allo-HSCT), the difference was not significant (*p* = 0.28; Fig. 2a). Similarly, the *S. maltophilia*-related 90-day mortality rate was nominally higher in allo-HSCT recipients compared to non-allo-HSCT recipients, but this difference was not statistically significant (34.0% [16/47] vs. 22.2% [4/18], respectively; *p* = 0.35; Fig. 2b).

**Table 1:** Comparison of patient characteristics between patients receiving allo-HSCT and patients receiving chemotherapy or auto-HSCT

		Allo-HSCT (*n* = 47)	Chemotherapy or auto-HSCT (*n* = 18)	*p*-value
Age	median, (range)	49 (19–70)	59 (21–77)	0.02
Sex	Male (%)	30 (64)	8 (44)	
	Female (%)	17 (36)	10 (56)	
Primary disease	AML	22 (46.8)	10 (55.6)	
	ALL	9 (19.1)	2 (11.1)	
	MDS	8 (17.0)	1 (5.6)	
	CML	3 (6.4)	0 (0.0)	
	AA	2 (4.3)	1 (5.6)	
	Aggressive NK leukemia	1 (2.1)	0 (0.0)	
	NHL	1 (2.1)	0 (0.0)	
	PMF	1 (2.1)	0 (0.0)	
	Myeloma	0 (0.0)	3 (16.7)	
Creatinine (mg/dl)	median, (range)	0.70 (0.3–3.0)	0.60 (0.3–6.3)	
CRP (mg/dl)	median, (range)	5.6 (0.3–40.8)	5.6 (0.2–34.0)	
Albumin (g/dl)	median, (range)	3.2 (1.6–4.2)	2.9 (2.10–4.20)	
Neutropenia (%)		31 (66.0)	13 (72.2)	
Profound neutropenia (%)		21 (44.7)	10 (55.6)	
Duration of neutropenia (days)	median, (range)	5.5 (0–30)	4.5 (1–42)	
Prolonged neutropenia (%)		14 (29.8)	6 (33.3)	
Initial source of infection (%)	Sources unknown	26 (55.3)	13 (72.2)	
	Cellulitis	3 (6.4)	1 (5.6)	
	CLABSI	13 (27.7)	4 (22.2)	
	Pneumonia	5 (10.6)	0 (0.0)	
Continuous bacteremia (%)		31 (66.0)	7 (38.9)	
Polymicrobial bacteremia (%)		19 (40.4)	5 (27.8)	
Severe sepsis or septic shock (%)		20 (42.6)	3 (16.7)	
Primary disease risk (%)^a^	High	25 (53.2)	9 (50.0)	
Central venous catheter (%)		45 (95.7)	11 (61.1)	<0.01
Mucositis (%)		20 (42.6)	1 (5.6)	<0.01
Diarrhea (%)		26 (55.3)	3 (16.7)	<0.01
Total parenteral nutrition (%)^b^		21 (44.7)	1 (5.6)	<0.01
Administration of insulin (%)^b^		13 (27.7)	0 (0.0)	0.01
Urine catheter (%)		12 (25.5)	1 (5.6)	
Past history of broad-antibiotics (%)^c^		34 (72.3)	14 (77.8)	
Past history of carbapenem (%)^c^		24 (51.1)	10 (55.6)	
Decade	2005 to 2009 (%)	14 (29.8)	6 (33.3)	
	2010 to 2014 (%)	33 (70.2)	12 (66.7)	
Time to appropriate therapy	median, (range)	3 (0–30)		
Source of transplantation (%)	BM	34 (72.3)		
	PB	7 (14.9)		
	CB	6 (12.8)		
Related or Unrelated donor (%)	Related donor	10 (21.3)		
	Unrelated donor	37 (78.7)		
HLA-matched donor (%)		22 (46.8)		
Conditioning intensity	Myeloablative	33 (70.2)		
	Reduced-intensity	14 (29.8)		
Acute GVHD (%)^d^		15 (31.9)		
Grade (%)	I	4 (26.7)		
	II	10 (66.7)		
	III	1 (6.7)		
Manifestation of acute GVHD^e^	Skin	14 (93.3)		
	Gut	4 (26.7)		
Onset of bacteremia	During conditioning	1 (2.1)		
	Before engraftment	31 (66.0)		
	After engraftment	15 (31.9)		
Days from transplantation (days)	median, (range)	18 (−6–1434)		

*Abbreviations*: *allo-HSCT* allogeneic hematopoietic stem cell transplantation, *auto-HSCT* autologous hematopoietic stem cell transplantation, *AML* acute myeloid leukemia, *ALL* acute lymphoblastic leukemia, *MDS* myelodysplastic syndrome, *CML* chronic myeloid leukemia, *CMML *chronic myelomonocytic leukemia, NHL non-Hodgkin lymphoma, *PMF* primary myelofibrosis, *AA* aplastic anemia, *CRP* c-reactive protein, *CLABSI* central-line associated blood stream infection, *GVHD* graft-versus-host disease, *BM* bone marrow, *PB* peripheral blood, *CB* cord blood, *HLA* human leukocyte antigen

The level of creatinine, CRP, albumin, and neutrophil count were applied from the point of onset of bacteremia

^a^Primary disease risk was classified into 2 categories; high-risk included acute leukemia not in remission, myelodysplastic syndrome with excess blast count or chronic myelomonocytic leukemia, chronic myeloid leukemia in blast crisis, the others were classified as standard-risk

^b^Total parenteral nutrition and insulin therapy were considered, if they were used within one week from the onset of bacteremia

^c^Broad-antibiotics included cefepime, piperacillin-tazobactam, and meropenem administered within past 30-days

^d^Acute GVHD was diagnosed and graded in accordance with previous reported consensus [35]

^e^Three patients had both skin and gut acute GVHD at the onset of bacteremia

Among the *S. maltophilia* strains isolated from patients, antimicrobial susceptibility to TMP-SMX, minocycline, levofloxacin, and ceftazidime was observed in 94%, 100%, 92%, and 40% of the strains, respectively. These frequencies were not significantly different between allo-HSCT recipients and patients not receiving allo-HSCT (data not shown).

Among allo-HSCT recipients, 8 out of 47 (17%) patients developed breakthrough *S. maltophilia* bacteremia. One of these 8 patients had received levofloxacin, and the other 7 of 8 were receiving TMP-SMX at the onset of *S. maltophilia* bacteremia. The probabilities of development of all-graded and graded II to IV acute GVHD at the onset of *S. maltophilia* bacteremia among 47 allo-HSCT recipients were 31.9% and 23.4%, respectively. Most of the patients with all-graded acute GVHD (14/15) had skin manifestations, and only 4 out of 15 patients had gut manifestations. Days from transplantation to onset of *S. maltophilia* bacteremia was 18 days (range, −6 to 1434), and 17 of the 47 (34.1%) allo-HSCT recipients developed *S. maltophilia* bacteremia after achievement of neutrophil engraftment.

### Risk factors for overall 90-day mortality in allo-HSCT recipients

**Fig. 3:**
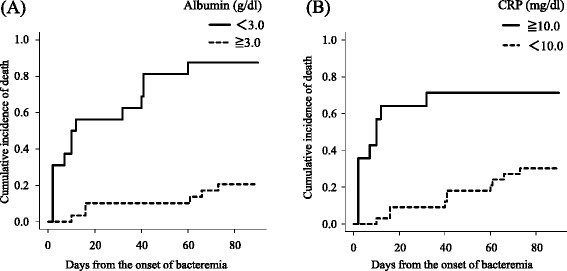
Overall mortality in allo-HSCT recipients stratified by the levels of serum albumin (**a**) and CRP (**b**) at the onset of bacteremia

Among 47 allo-HSCT recipients, univariate analysis showed that high levels of serum CRP (≥10.0 mg/dl), low levels of serum albumin (<3.0 g/dl), increased serum creatinine levels (≥1.0 mg/dl), high disease risk, severe sepsis or septic shock, initial source of infection, time to appropriate therapy, non-removal of CVC, and reduced-intensity conditioning regimens were significant risk factors for overall 90-day mortality. Independent risk factors associated with overall 90-day mortality were high levels of serum CRP (adjusted hazard ratio [aHR], 3.28; 95% CI, 1.00–10.72; *p* = 0.05) and low levels of serum albumin (aHR, 10.86; 95% CI, 3.27–36.12; *p* < 0.01) (Table 2). Figure 3 shows overall 90-day mortality stratified by levels of serum albumin and CRP at the onset of bacteremia. Overall 90-day mortalities in patients with or without low levels of albumin were 87.5% and 20.7%, respectively (*p* < 0.01), and those in patients with or without high levels of CRP were 71.4% and 30.3%, respectively (*p* < 0.01). The distribution of the values of albumin and CRP, and the correlation of the values of albumin or CRP and survival days are shown in Additional file 2: Figure S2 and Additional file 3: Figure S3, respectively.

**Table 2:** Risk factors for overall 90-day mortality in allo-HSCT recipients with *S. maltophilia* bacteremia

	Univariate analysis			Multivariate analysis		
		Probability of survival	*p*-value	Hazard ratio	95% CI	*p*-value
Age (years)	≥60	0.63 (0.45–0.77)	0.13			
	<60	0.42 (0.15–0.67)				
Sex	Male	0.60 (0.41–0.75)	0.67			
	Female	0.53 (0.28–0.73)				
CRP (mg/dl)	≥10	0.29 (0.09–0.52)	<0.01	3.28	1.00–10.72	0.05
	<10	0.70 (0.51–0.82)				
Albumin (g/dl)	<3.0	0.13 (0.02–0.33)	<0.01	10.86	3.27–36.12	<0.01
	≥3.0	0.79 (0.59–0.90)				
Creatinine (mg/dl)	≥1.0	0.25 (0.06–0.51)	<0.01			
	<1.0	0.69 (0.51–0.81)				
Neutropenia (≤500 /μl)	Yes	0.55 (0.36–0.70)	0.52			
	No	0.63 (0.35–0.81)				
Profound neutropenia (≤100 /μl)	Yes	0.48 (0.26–0.67)	0.1			
	No	0.65 (0.44–0.80)				
Prolonged neutropenia (≥7 days)	Yes	0.79 (0.47–0.93)	0.08			
	No	0.49 (0.31–0.64)				
Onset of bacteremia	During conditioning	1.00 (1.00–1.00)	0.73			
	Before engraftment	0.60 (0.32–0.80)				
	After engraftment	0.55 (0.36–0.70)				
Existence of acute GVHD	Yes	0.47 (0.21–0.69)	0.44			
	No	0.63 (0.44–0.77)				
Diarrhea	Yes	0.62 (0.40–0.77)	0.64			
	No	0.52 (0.30–0.71)				
Mucositis	Yes	0.60 (0.36–0.78)	0.89			
	No	0.56 (0.35–0.72)				
Primary disease risk	High	0.40 (0.21–0.58)	<0.01			
	Standard	0.77 (0.54–0.89)				
Polymicrobial bacteremia	Yes	0.58 (0.33–0.76)	0.88			
	No	0.57 (0.37–0.73)				
Severe sepsis or septic shock	Yes	0.30 (0.12–0.50)	<0.01			
	No	0.78 (0.57–0.89)				
Initial source of infection	Sources unknown	0.58 (0.37–0.74)	<0.01			
	Cellulitis	1.00 (1.00–1.00)				
	CLABSI	0.69 (0.37–0.87)				
	Pneumonia	0.00 (NA)				
Time to appropriate therapy (days)	≥4	0.76 (0.54–0.88)	0.05			
	≤3	0.47 (0.23–0.68)				
Removal of CVC	Yes	0.92 (0.57–0.99)	<0.01			
	No	0.44 (0.27–0.60)				
Past history of carbapenem	Yes	0.46 (0.26–0.64)	0.05			
	No	0.70 (0.47–0.84)				
Source of transplantation	BM	0.62 (0.43–0.76)	0.17			
	PB	0.57 (0.17–0.84)				
	CB	0.33 (0.05–0.68)				
Donor type	Related	0.80 (0.41–0.95)	0.1			
	Unrelated	0.51 (0.34–0.66)				
HLA-matching	Match	0.64 (0.40–0.79)	0.38			
	Mismatch	0.52 (0.31–0.69)				
Conditioning	Myeloablative	0.73 (0.54–0.85)	<0.01			
	Reduced-intensity	0.21 (0.05–0.45)				
Study period	2005–2009	0.57 (0.28–0.78)	0.95			
	2010–2014	0.58 (0.39–0.72)				

*Abbreviations*: *Allo-HSCT* allogeneic hematopoietic stem cell transplantation, *S. maltophilia Stenotrophomonas maltophilia*, *CI* confedence interval, *CRP* c-reactive protein, *GVHD* graft-versus-host disease, *CLABSI* central-line associated blood stream infection, *CVC* central venous catheter, *NA* not available, *BM* bone marrow, *PB* peripheral blood, *CB* cord blood, *HLA* human leukocyte antigen

Details for patients with low levels of albumin and high levels of CRP are shown in Table 3. Among 9 recipients with both high CRP and low albumin levels, 4 recipients had neutrophil counts equal to or exceeding 500/μl at the onset of bacteremia, and 8 recipients had severe sepsis or septic shock. Although 5 recipients had pneumonia at the onset of bacteremia, the remaining 4 recipients developed pneumonia, even under effective treatments, in a median of 3 days (range, 1 to 8 days). All 9 patients eventually died in a median of 2 days (range, 2 to 32 days), and all of these deaths were attributable to *S. maltophilia* bacteremia. One individual (Patient No. 9) survived 32 days from the onset of bacteremia after receiving a combination therapy of tigecycline (TGC) and TMP-SMX. The probabilities of developing pneumonia in patients with or without high CRP and low albumin levels were 100% (9/9) and 10.5% (4/38), respectively (*p* < 0.01).

**Table 3:** Details of 9 patients with low albumin and high CRP

No.	Age	Primary disease	Stem cell source	Conditioning	Duration from transplantation to infection (days)	Engraftment	CRP (mg/dl)	Albumin (g/dl)	Severe sepsis or septic shock	Initial source of infection	Subsequent progression to pneumonia (days)	Treatment	Survival (days)
1	32	AML	uBM	BU+CY+TLI	124	Yes	11.6	1.9	-	Sources unknown	Yes (1)	MINO	12
2	58	AML	CB	Flu+Mel+TBI	26	No	14.4	2.3	Severe	Pneumonia		-	2
3	54	AML	CB	CA+CY+TBI	15	Yes	18.5	2.5	Severe	Sources unknown	Yes (5)	MINO	7
4	70	CMLBC	CB	Flu+Mel+TBI	20	No	40.8	2.9	Severe	Pneumonia		CPFX	2
5	69	AML	uBM	Flu+Mel+TBI	11	Yes	19.8	2.6	Shock	Pneumonia		-	2
6	64	PMF	uBM	Flu+Mel+TBI	34	No	25.3	1.6	Severe	Pneumonia		CPFX	2
7	57	FL	uPB	Flu+CY+TBI	10	No	15.9	2.4	Shock	Sources unknown	Yes (8)	CPFX+MINO	10
8	30	AML	uBM	CY+TBI	373	Yes	23	1.6	Shock	CLABSI	Yes (1)	CPFX+MINO→ST+MINO	2
9	56	AML	uBM	Flu+BU+TBI	17	No	17.8	1.6	Severe	Pneumonia and cellulitis		TGC→ST+TGC	32

*Abbreviations*: *AML* acute myeloid leukemia, *CMLBC* chronic myeloid leukemia blast crisis, *PMF* primary myelofibrosis, *FL* follicular lymphoma, *uBM* unrelated-bone marrow, *CB* cord blood, *uPB* unrelated-peripheral blood, *BU* busulfan, *CY* cyclophosphamide, *TLI* total lymphoid irradiation, *Flu* fludarabine, *Mel* melphalan, *TBI* total body irradiation, *CRP* c-reactive protein, *CLABSI* central-line associated blood stream infection, *MINO* minocycline, *CPFX* ciprofloxacin, *ST* trimethoprim-sulfamethoxazole, *TGC* tigecycline

### Overall 90-day mortality based on antimicrobial regimen

Allo-HSCT recipients with *S. maltophilia* bacteremia received various combination therapies or monotherapies, which consisted of ciprofloxacin, levofloxacin, TMP-SMX, minocycline, TGC, or the combination thereof. The overall 90-day mortality rate for the antimicrobial regimens did not differ significantly (39.4% with TMP-SMX or minocycline, 66.7% with ciprofloxacin or levofloxacin, and 37.5% without any effective antibiotic; *p* = 0.42).

The rate of receiving effective antimicrobials within 3 days from the onset of bacteremia was 42.6%. Landmark analysis showed that there was no significant difference in overall 90-day mortality between patients with or without receiving effective antimicrobials within 3 days from the onset (42.9% vs. 26.3%, respectively; *p* = 0.16*)*, even among recipients with high CRP or low albumin levels (37.5% vs. 37.5%, respectively; *p* = 0.74).

## Discussion

In the present study, we showed a higher incidence of *S. maltophilia* bacteremia in allo-HSCT recipients than in patients not receiving allo-HSCT. Low levels of albumin and high levels of CRP were found to be risk factors for overall 90-day mortality in allo-HSCT recipients. Additionally, all patients with low albumin and high CRP levels developed pneumonia initially or subsequently, even with the administration of effective antimicrobials, and subsequently died.

The present study indicated a higher incidence of *S. maltophilia* bacteremia in allo-HSCT recipients. Yeshurun et al. reported a higher incidence of *S. maltophilia* bacteremia in allo-HSCT recipients compared with auto-HSCT recipients (5.6% vs. 1%, respectively) [10]. However, their report included only 19 episodes of bacteremia over four years. Other study showed that the incidence of *S. maltophilia* bacteremia was 1.34 cases per 10,000 patient-days among patients with hematological malignancy [11]. Although these incidences were apparently lower, we think the differences could mainly be attributed to differences in patient characteristics. First, the rate of the patients receiving allo-HSCT in our study was more than three-fold higher compared to that in the study (72% vs. 23%, respectively). Second, allo-HSCT recipients were younger, and more likely to have diarrhea, mucositis, CVC, total parenteral nutrition, and/or insulin therapy. Most of these factors are considered as predisposing risk factors for *S. maltophilia* bacteremia based on the results of past studies [3, 7–9]. Notably, we saw no evidence or report of horizontal spread of *S. maltophilia* during our study; this lack presumably reflected strict monitoring for rapid detection of outbreaks through daily investigations by an infection control team at our facility.

Our study also demonstrated the increasing incidence of *S. maltophilia* bacteremia over the last 5 years, a pattern that is consistent with those of previous studies [4, 12, 22]. This elevated incidence of *S. maltophilia* bacteremia may reflect the increasing use of more profound immunosuppressive agents for prevention and treatment of GVHD in the last 5 years; the rate of additional immunosuppressive agents other than calcineurin inhibitor rose from 35.7% in the early period to 60.6% in the later period. The effect may also reflect increasing frequencies of HSCT in high-risk patients, with the procedure now being employed in older patients (median ages rising from 41 years in the earlier period to 53 years in the later period) and in patients with active primary diseases (42.9% vs. 57.6%, respectively).

The overall mortality rate of *S. maltophilia* bacteremia in the present study (42.6%) was comparable to that obtained in past studies, where reported rates ranged from 21 to 64.5% [7, 8, 11, 23]. As noted above, the characteristics of allo-HSCT recipients differed significantly from those of patients not receiving allo-HSCT. Therefore, it is important to clarify risk factors for mortality specific to allo-HSCT recipients. In the present study, high CRP levels and low albumin levels were found to be risk factors for overall 90-day mortality in allo-HSCT recipients. Although CRP is an acute-phase protein which increases in response to infection, its prognostic value has been evaluated in various settings [24–26]. Serum albumin is also one of the negative acute-phase proteins as well as an indicator of previous malnutrition. Several reports showed that serum albumin levels were associated with prognosis in pneumonia, severe sepsis, and bacteremia [26–28]. Additionally, it was demonstrated that a combination of CRP and albumin levels was better prognostic marker rather than either parameter alone [29, 30]. Tada et al. [7] reported that high serum CRP levels were associated with *S. maltophilia* hemorrhagic pneumonia, known to be one of the most fatal forms of *S. maltophilia* infection. That study revealed 100% mortality among 7 patients with hemorrhagic pneumonia due to *S. maltophilia*. In addition, Mori et al. reported 30 episodes of hemorrhagic pneumonia caused by *S. maltophilia*, and those authors also noted detrimental outcomes, yielding 100% mortality [31]. Our results are consistent with those studies, and the combination of high CRP and low albumin levels appears to readily predict the prognosis of *S. maltophilia* bacteremia. Not only did all 5 patients having pneumonia at the onset of bacteremia die, but also all 4 patients who subsequently developed pneumonia during the treatment of bacteremia followed a fatal clinical course, despite receiving effective antimicrobial therapies. If allo-HSCT recipients with *S. maltophilia* bacteremia have respiratory symptoms (such as cough, sputum, chest pain, and desaturations) or have risk factors as above, clinicians should consider the possibility of hemorrhagic pneumonia by *S. maltophilia* or subsequent progression to pneumonia. When a sputum culture is ordered, the result of Gram staining would be a clue for rapid detection of pneumonia.

Levofloxacin and TGC had recently been proposed as alternative options beyond TMP-SMX in the treatment of *S. maltophilia* infections [32, 33]. In cases involving allo-HSCT recipients, clinicians might hesitate to administer TMP-SMX due to hematologic toxicity, especially during the pre-engraftment period. Our study demonstrated the fatal outcomes of allo-HSCT recipients with low albumin and high CRP levels when such patients were treated empirically with ciprofloxacin or minocycline. Although we did not observe significant differences in outcome among patients treated with of TMP-SMX, minocycline, and fluoroquinolones, this lack of distinction likely was due to treatment selection bias and a lack of statistical power. The Fourth European Conference on Infections in Leukemia recommended combination therapy with TMP-SMX in seriously ill or neutropenic patients with *S. maltophilia* infection, however, further studies are warranted [34].

There are some limitations to this study. First, the present study was a single-center retrospective cohort study. As such, this work may have underestimated the incidence and mortality for *S. maltophilia* bacteremia, and may have misclassified the source of bacteremia. Second, the number of patients was limited due to the low incidence of *S. maltophilia* bacteremia, which could have led to underpowered analyses. Third, treatment selection bias may have influenced the apparent mortality rate. However, few studies on risk factors for mortality in *S. maltophilia* bacteremia following allo-HSCT have been reported. The present study is worth reporting because our results are expected to help clinicians employ easily available tests to identify patients who may harbor risk factors for mortality.

## Conclusions

The incidence of *S. maltophilia* bacteremia was higher in allo-HSCT recipients than in patients not receiving allo-HSCT. Among allo-HSCT recipients with *S. maltophilia* bacteremia, low albumin and high CRP levels appear to be predictive of disease progression to pneumonia and poor prognosis. Clinicians should consider those possibilities, perform appropriate tests, and promptly initiate antimicrobial treatment.

## Additional files

Additional file 1: Figure S1. The Receiver Operating Characteristics curve analysis for the cut-off value of albumin (a and b) and CRP (c and d). (PPTX 50 kb)

Additional file 2: Figure S2. The distributions of the values of albumin and CRP. (PPTX 80 kb)

Additional file 3: Figure S3. The correlation between survival days and the value of albumin (a) or CRP (b). Patients who survived more than 90 days were censored at the day 90. (PPTX 41 kb)

## Abbreviations

allo-HSCT: allogeneic hematopoietic stem cell transplantation
ANC: absolute neutrophil count
auto-HSCT: autologous hematopoietic stem cell transplantation
CLABSI: central line-associated blood stream infection
CRP: C-reactive protein
CVC: central venous catheters
GVHD: graft-vs.-host disease
HLA: human leukocyte antigen
IDSA: Infectious Diseases Society of America
*S. matophilia*: *Stenotrophomonas maltophilia*
SSCG: surviving sepsis campaign guidelines
TGC: tigecycline
TMP-SMX: trimethoprim-sulfamethoxazole
