# Supplementary figures and images for: Predictive implications of albumin and C-reactive protein for progression to pneumonia and poor prognosis in Stenotrophomonas maltophilia bacteremia following allogeneic hematopoietic stem cell transplantation
Source: BMC Infect Dis. 2017 Sep 22;17:638. doi: 10.1186/s12879-017-2745-6 (PMC5610439; doi:10.1186/s12879-017-2745-6)

## Slide 1
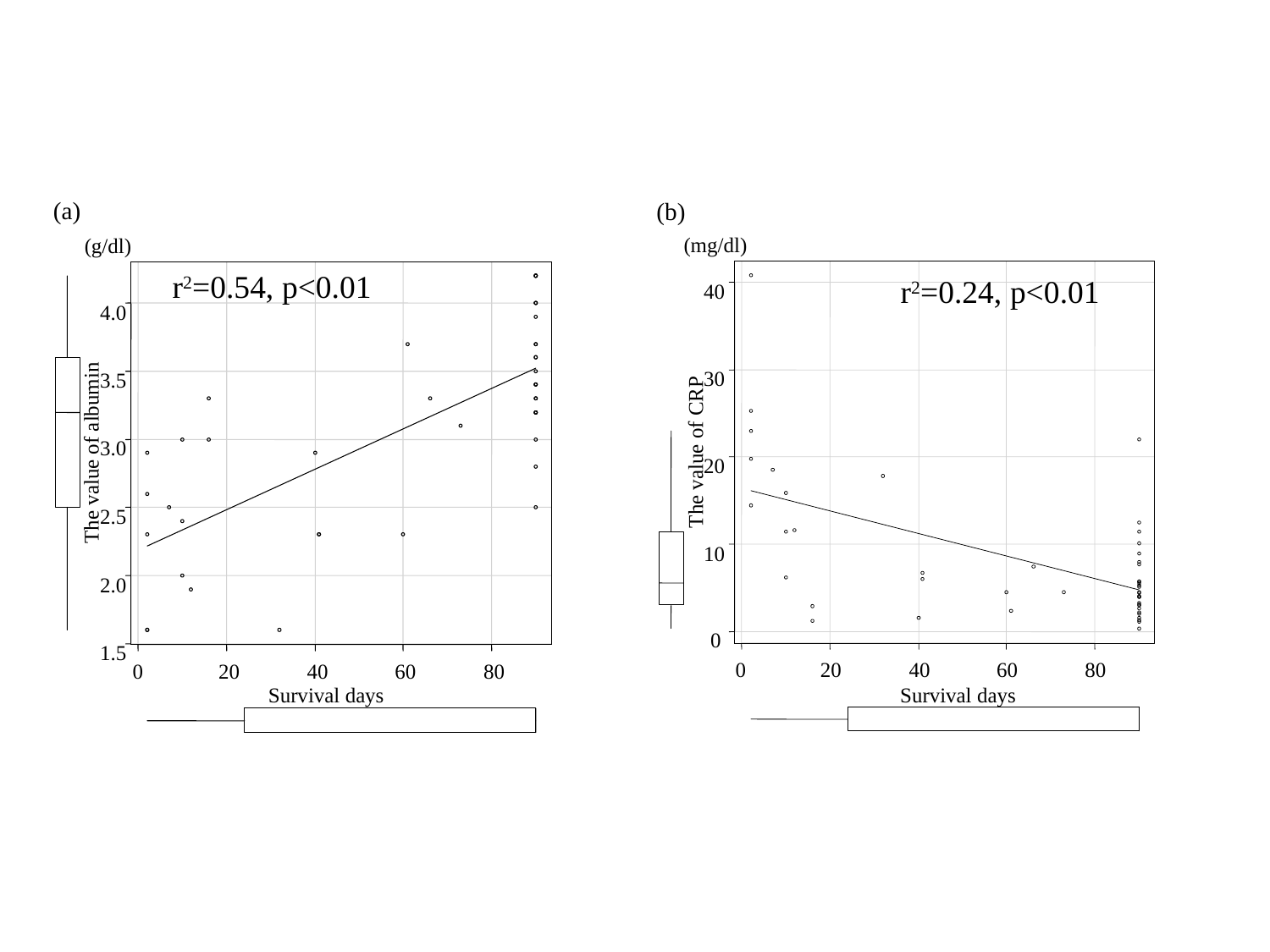

(a)
(b)
(mg/dl)
r2=0.54, p<0.01
4.0
3.5
3.0
The value of albumin
2.5
2.0
1.5
0
20
40
60
80
Survival days
(g/dl)
40
30
The value of CRP
20
10
0
0
20
40
60
80
Survival days
r2=0.24, p<0.01

Supplement: Supplementary file 1 — The Receiver Operating Characteristics curve analysis for the cut-off value of albumin (a and b) and CRP (c and d). (PPTX 50 kb) [file 12879_2017_2745_MOESM1_ESM.pptx]

## Slide 1
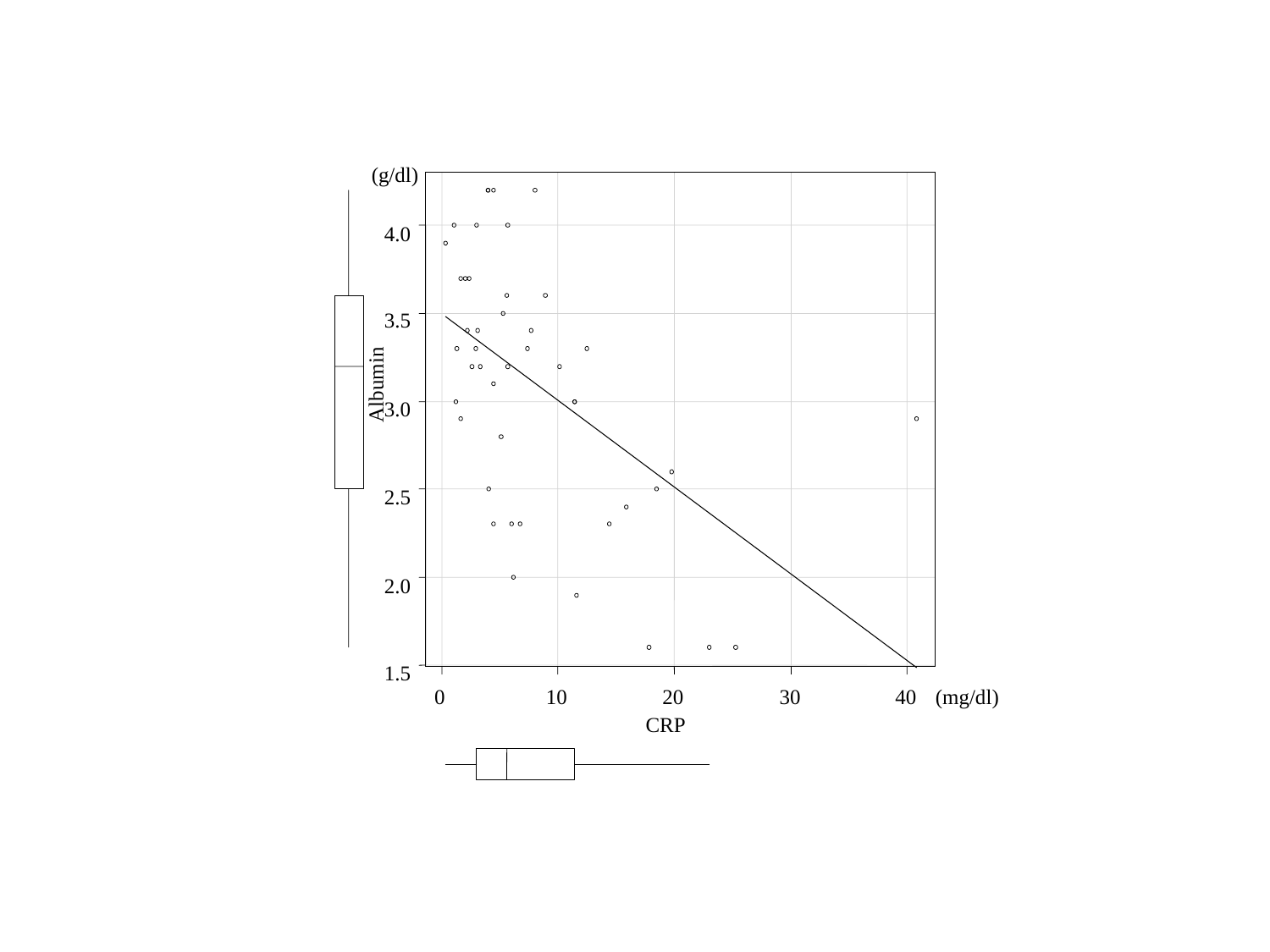

(g/dl)
4.0
3.5
Albumin
3.0
2.5
2.0
1.5
0
10
20
30
40
CRP
(mg/dl)

Supplement: Supplementary file 3 — The correlation between survival days and the value of albumin (a) or CRP (b). Patients who survived more than 90 days were censored at the day 90. (PPTX 41 kb) [file 12879_2017_2745_MOESM3_ESM.pptx]
