# Supplementary material for: Predictive implications of albumin and C-reactive protein for progression to pneumonia and poor prognosis in Stenotrophomonas maltophilia bacteremia following allogeneic hematopoietic stem cell transplantation
Source: BMC Infect Dis. 2017 Sep 22;17:638. doi: 10.1186/s12879-017-2745-6 (PMC5610439; doi:10.1186/s12879-017-2745-6)
Supplement: Supplementary file 2 — The distributions of the values of albumin and CRP. (PPTX 80 kb) [file 12879_2017_2745_MOESM2_ESM.pptx]

## Slide 1
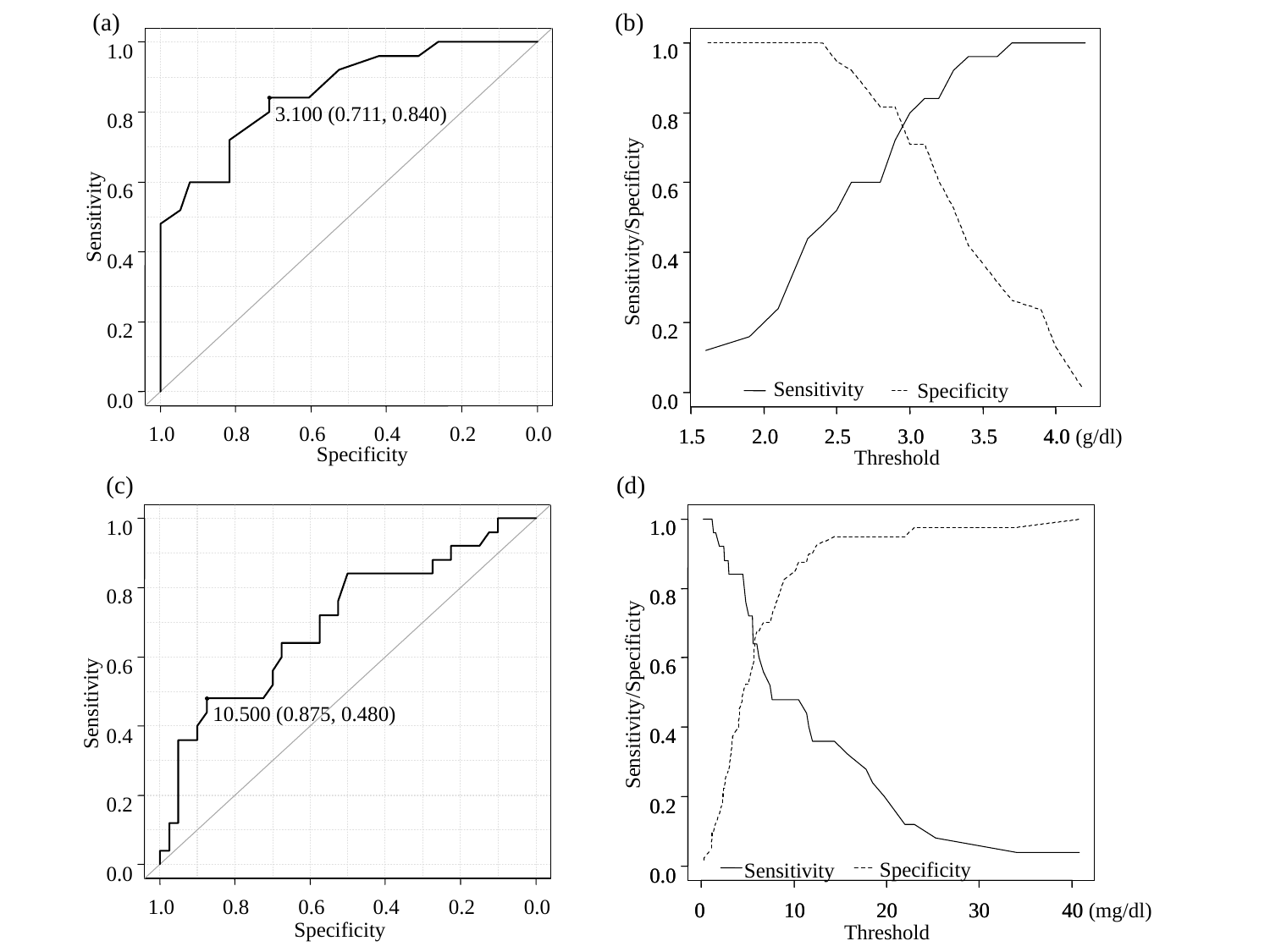

(a)
(b)
1.0
3.100 (0.711, 0.840)
0.8
0.6
Sensitivity
0.4
0.2
0.0
1.0
0.8
0.6
0.4
0.2
0.0
Specificity
1.0
1.0
0.8
0.8
0.6
0.6
Sensitivity/Specificity
0.4
0.4
0.2
0.2
Sensitivity
Specificity
0.0
0.0
1.5
1.5
2.0
2.0
2.5
2.5
3.0
3.0
3.5
3.5
4.0
4.0 (g/dl)
Threshold
(c)
(d)
1.0
0.8
0.6
Sensitivity
10.500 (0.875, 0.480)
0.4
0.2
0.0
1.0
0.8
0.6
0.4
0.2
0.0
Specificity
1.0
1.0
0.8
0.8
0.6
0.6
Sensitivity/Specificity
0.4
0.4
0.2
0.2
Specificity
Sensitivity
0.0
0.0
0
10
20
30
40 (mg/dl)
0
10
20
30
40
Threshold
